# Serotonin Receptor Expression in Human Prefrontal Cortex: Balancing Excitation and Inhibition across Postnatal Development

**DOI:** 10.1371/journal.pone.0022799

**Published:** 2011-07-29

**Authors:** Evelyn K. Lambe, Stu G. Fillman, Maree J. Webster, Cynthia Shannon Weickert

**Affiliations:** 1 Department of Physiology, University of Toronto, Toronto, Ontario, Canada; 2 Department of Obstetrics and Gynaecology, University of Toronto, Toronto, Ontario, Canada; 3 Schizophrenia Research Institute, Sydney, New South Wales, Australia; 4 Schizophrenia Research Laboratory, Neuroscience Research Australia, Randwick, New South Wales, Australia; 5 Faculty of Medicine, School of Psychiatry, University of New South Wales, Sydney, New South Wales, Australia; 6 Stanley Medical Research Institute, Rockville, Maryland, United States of America; Institut National de la Santé et de la Recherche Médicale, France

## Abstract

Serotonin and its receptors (*HTRs*) play critical roles in brain development and in the regulation of cognition, mood, and anxiety. *HTRs* are highly expressed in human prefrontal cortex and exert control over prefrontal excitability. The serotonin system is a key treatment target for several psychiatric disorders; however, the effectiveness of these drugs varies according to age. Despite strong evidence for developmental changes in prefrontal *Htrs* of rodents, the developmental regulation of *HTR* expression in human prefrontal cortex has not been examined. Using *postmortem* human prefrontal brain tissue from across postnatal life, we investigated the expression of key serotonin receptors with distinct inhibitory (*HTR1A*, *HTR5A*) and excitatory (*HTR2A, HTR2C*, *HTR4*, *HTR6*) effects on cortical neurons, including two receptors which appear to be expressed to a greater degree in inhibitory interneurons of cerebral cortex (*HTR2C, HTR6*). We found distinct developmental patterns of expression for each of these six *HTRs*, with profound changes in expression occurring early in postnatal development and also into adulthood. However, a collective look at these *HTRs* in terms of their likely neurophysiological effects and major cellular localization leads to a model that suggests developmental changes in expression of these individual *HTR*s may not perturb an overall balance between inhibitory and excitatory effects. Examining and understanding the healthy balance is critical to appreciate how abnormal expression of an individual *HTR* may create a window of vulnerability for the emergence of psychiatric illness.

## Introduction

In the adult, modulation of the prefrontal cortex by the neuromodulator serotonin is critical for emotional regulation and resilience to stress [1], [2], [3], [4]. The prefrontal cortex is a late maturing brain region [5], [6], [7], [8], [9] with an extensive interrelationship with the serotonin system [10], [11], [12]. Although the serotonergic innervation of the prefrontal cortex matures early in primate postnatal brain development [13], its effects on the developmental neurophysiology and growth of the human prefrontal cortex are much less well understood. Medicines that target the serotonin system are used to treat symptoms of anxiety or depression but appear to be less effective during childhood and have an increased risk of adverse effects when administered to children as compared to adults [14], [15]. In fact, recent work suggests that exposure to serotonergic medicines in development increases behaviours suggestive of greater anxiety and sensitivity to stress [16], [17].

Serotonin has been implicated as a trophic factor in brain development [18], [19], [20], [21], and its effects show significant developmental changes [22], [23]. It is thought that these developmental changes in the functional and pharmacological effects of serotonin are due to changes in the expression of individual postsynaptic receptors [22], [24]. In humans, developmental changes in the expression of serotonin receptors (*HTRs*) would alter the functional effects of serotonin and serotonergic medicines, yet developmental changes in *HTRs* have not been systematically examined in human prefrontal cortex.

Postsynaptic serotonin receptors control how the prefrontal cortex responds to serotonin at baseline and to the increased release of serotonin during stress [1]. The most well-studied receptors, *HTR1A* and *HTR2A*, are highly expressed in excitatory neurons of prefrontal cortex [25], [26], [27], [28]. Under baseline conditions, endogenous serotonin release triggers an *Htr1A*-mediated inhibition of prefrontal cortex [11], [29], [30] with *Htr2A*-mediated effects potentially recruited at higher levels of serotonin release [25], [30]. Behaviourally, these two serotonin receptors in adult prefrontal cortex appear to have opposite effects on anxiety and mood, with high levels of the inhibitory *HTR1* being anxiolytic [31], [32], the excitatory *Htr2A* being required for normal anxiety levels [33] and higher *HTR2A* levels being associated with mood disturbance [34], [35]. Work in rodents suggests that there is a striking developmental change in the functional balance of *Htr1A* and *Htr2A* expression [22], [23]; this relationship has not yet been examined in the developing human prefrontal cortex.

Beyond the well-studied *HTR1A* and *HTR2A*, the serotonin receptor family is large, consisting of 14 members in total. Little is known about the human developmental trajectories of all the serotonin receptors expressed in prefrontal cortex. In this work, we examine *HTR1A* and *HTR2A* together with a selection of the other receptors, including *HTR2C*, *HTR4*, *HTR5A*, and *HTR6*. The excitatory *Htr2C* receptor appears to be more strongly expressed in cortical interneurons [36], [37], [38] and plays a critical role within prefrontal cortex in controlling impulsivity [39], [40]. The inhibitory *Htr5A* receptor is expressed in a significant portion of cortical pyramidal neurons [41], [42], [43], [44] and may influence anxiety levels under stress [45]. The excitatory *Htr4* and *Htr6* are thought to be expressed respectively in pyramidal neurons [22], [46], [47] and interneurons [48], [49] and are both important in cognition [50], [51].

We examine the developmental changes in the expression of these serotonin receptors in human prefrontal cortex from infancy to adulthood. Since serotonin receptors comprise a large family with diverse localization and functions, we have chosen six receptors with a variety of expression patterns and neurophysiological coupling. We have also made particular effort to relate developmental changes in the *HTRs* expression to developmental changes in inhibitory interneuron markers in order to determine if changes in serotonin neurotransmission may be synchronized with the maturation of interneuron subtypes. This human developmental information is important in order to appreciate vulnerable time periods in the postnatal prefrontal cortex and to gain insight into potential mechanisms underlying changes in the effects of serotonin and serotonergic medicines.

## Materials and Methods

### Human Postmortem Brain Samples

Human *postmortem* tissue from the dorsolateral prefrontal cortex was obtained from the NICHD Brain and Tissue Bank for Developmental Disorders at the University of Maryland, Baltimore, MD, USA (contract HHSN275200900011C, Ref. No. N01-HD-9-0011). Written consent was obtained from individuals or their next of kin before tissue donation. Samples were obtained from 59 individuals who ranged in age from six weeks to 49 years and were grouped into seven developmental periods: neonates (n = 8), infants (n = 13), toddlers (n = 7), school age (n = 7), teenagers (n = 7), young adults (n = 9) and adults (n = 8). Demographic details and sample characteristics are summarised in **Table 1** with full details available in **Table S1**. Sample preparation for mRNA expression analyses have been described previously [52]. This study was carried out in accordance with the latest version of the Declaration of Helsinki after specific approval by the University of NSW Human Research Ethics Committee (HREC # 07261).

**Table 1:** Demographics of developmental subjects in the Maryland Brain Bank *postmortem* tissue cohort.

Group	Age (years)	PMI (hours)	Gender	pH	Race	RIN
Neonate	0.17 (0.11*–*0.24)	22.5±6.0	5M : 3F	6.60±0.14	6AA : 2C	8.01±0.50
Infant	0.53 (0.25*–*0.91)	17.5±6.4	8M : 5F	6.61±0.16	10AA : 3C	7.56±0.92
Toddler	2.51 (1.58*–*4.64)	19.2±5.8	3M : 4F	6.77±0.17	4AA : 3C	7.15±0.72
School Age	9.98 (7.84*–*12.98)	14.4±5.2	3M : 4F	6.70±0.21	2AA : 5C	7.54±0.58
Teenage	16.8 (16.34*–*17.82)	17.9±3.9	5M : 2F	6.74±0.08	1AA : 6C	6.98±0.86
Young Adult	23.2 (20.14*–*25.38)	13.7±8.3	5M : 4F	6.67±0.23	5AA : 4C	7.46±0.97
Adult	43.4 (35.99*–*48.7)	13.4±4.6	5M : 3F	6.60±0.27	4AA : 4C	7.16±0.93
*All Ages*	*13.0 (0.11–48.7)*	*16.9*±*6.4*	*34M : 25F*	*6.66*±*0.19*	*32AA : 27C*	*7.43*±*0.84*

All values ± standard deviation, PMI – post-mortem interval, RIN – RNA integrity number, AA - African American, C – Caucasian.

### Quantitative Real Time RT-PCR Analysis

*HTR* transcript expression levels were assessed using quantitative real-time RT-PCR (qPCR). cDNA was synthesized from total RNA (3 µg) of the entire cohort individuals using SuperScript**®** First-Strand Synthesis kit and random hexamers following the manufacturer's instructions (Invitrogen, Carlsbad, CA USA). The transcript levels for six genes of interest and four housekeeper genes were measured using an ABI Prism 7900HT Fast Real time PCR system with a 384-well format and TaqMan Gene Expression Assays (Applied Biosystems) (*HTR1A*: Hs00265014_s1, *HTR2A*: Hs01033524_m1, *HTR2C*: Hs00168365_m1, *HTR4*: Hs00168380_m1, *HTR5A*: Hs00225153_m1, *HTR6*: Hs00168381_m1, *GUSB*: Hs99999908_m1, *HMBS*: Hs00609297_m1, *PPIA*: Hs99999904_m1 and *UBC*: Hs00824723_m1 ). Cycling conditions and reaction amounts were as previously described [53].

### Gene Expression Normalization

In order to reduce the variability inherent in measuring mRNA from this number of individuals the data was normalized through the use of housekeeping genes. We chose four —β-glucuronidase (*GUSB*), hydroxymethylbilanesynthase (*HMBS*), peptidylprolylisomerase A (*PPIA*), and ubiquitin C (*UBC*) — which did not change through development (ANOVA, *GUSB*; F(6,61)  = 0.5 , p = 0.8, *HMBS*; F(6,61)  = 0.5, p = 0.7, *PPIA*; F(6,60)  = 0.4, p = 0.9, *UBC*; F(6,61)  = 0.08, p = 0.7). The stability of the expression of these genes was assessed through the use of the geNorm program which calculates the average pair-wise variation of a particular gene with all other control genes yielding an M-Value with lower number indicating greater stability (*GUSB*: 0.871, *HMBS*: 0.811, *PPIA*: 0.876, *UBC*: 0.923) [54]. We have avoided ribosomal mRNAs, which are expressed at very high levels, and instead chose two high expression and two medium expression genes. The chosen housekeepers are involved in the varied biological processes of the cell following the criteria previously established [54]. The housekeeping genes were averaged through the use of a geometric mean which was then used to normalize the *HTR* gene expression data.

### Statistical Analysis

**Figure 1:**
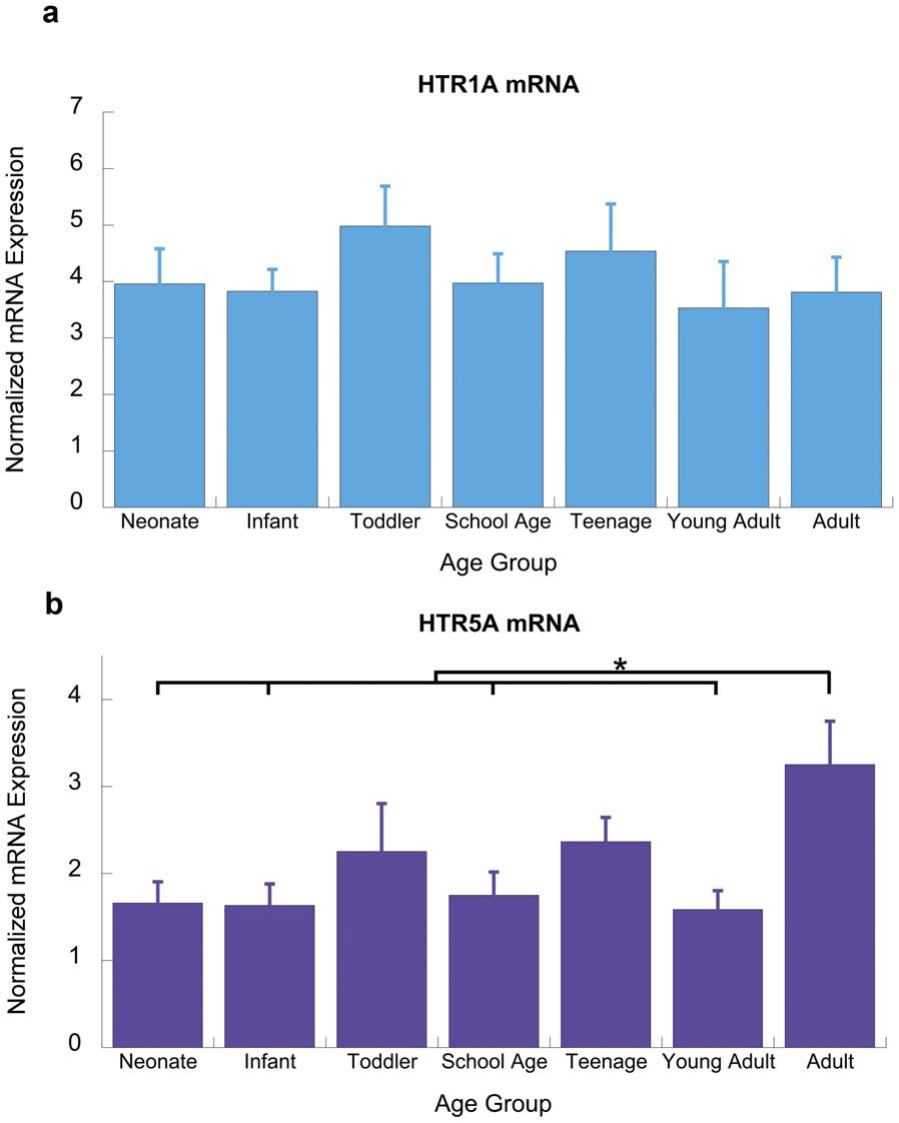
Developmental pattern of expression of Gα_i_-coupled serotonin receptors: *HTR1A* and *HTR5A*. (a) Normalized qPCR expression for *HTR1A* in human prefrontal cortex across postnatal development. The expression of *HTR1A* does not significantly change across the developmental groups (F(6,53)  = 0.6, p = 0.8). (b) Normalized qPCR expression for *HTR5A* in human prefrontal cortex across postnatal development. There is a significant developmental change in *HTR5A* expression (F(6,43) = 2.9, p<0.02), Asterisks show the significance levels of an ANOVA post-hoc Fisher LSD test (* p<0.05).

Population outliers were identified for each transcript measured through qPCR using the Grubb's test (*p<0.05*), resulting in the removal of the following individuals: one toddler from *HTR2C,* one young adult and one adult from *HTR4*, one young adult from *HTR5A,* and two infants and an adult from *HTR6*. All data were normally distributed (Lilliefors) and showed homogeneity of variance (Levene's, *p>0.20*). Statistical tests were performed using PASW statistics (version 18 for MacOSX, SPSS, Chicago, Il, USA) and included one-way analysis of variance (ANOVA) with a post hoc Fisher LSD to determine significant changes in gene expression between groups. Using normalized qPCR data, Pearson correlations were performed between the genes of interest and the brain sample characteristics (pH, RIN, PMI) with significant correlations being co-varied in an Analysis of Covariance (ANCOVA). For **Figure 1b** and to calculate the relative balance between *HTRs* with excitatory and inhibitory contributions, the measurements for *HTR5A* were multiplied by 10 to correct for the difference in cDNA dilution used in detection. Only individuals who had qPCR measurements from all six *HTRs* were included in the ANOVA to assess for changes in the expression ratio of excitatory and inhibitory receptors (neonate = 8, infant = 11, toddler = 6, school age = 7, teenage = 7, young adult = 6, adult = 6). Identifying significant linkages between the mRNA expression of the *HTRs* expressed by interneurons and other interneuron markers was done using a stepwise regression analysis and the interneuron marker mRNA expression data from Fung et al. [7]. This method allowed us to include only interneuron markers whose developmental expression patterns are significantly associated with developmental changes in individual *HTR* mRNA expression.

## Results

To investigate developmental changes in the expression of a subset of important serotonin receptors in human prefrontal cortex, we performed qPCR on a large sample of *postmortem* brains from infancy to adulthood. The *HTRs* we targeted include the two major serotonin receptors expressed in the prefrontal cortex, *HTR1A* and *HTR2A*, as well as *HTR2C*, *HTR4*, *HTR5A*, *HTR6*. As illustrated in **Table 2**, these receptors include representatives of all three classes of G-protein coupling (Gα_i_, Gα_q_, and Gα_s_), which have distinct effects on cellular excitability. However, to appreciate the potential net overall effect of each receptor on the excitability of the prefrontal cortex, this receptor coupling must be viewed in the context of cellular localization. As illustrated in **Table 3**, some *HTRs* are predominantly expressed by excitatory pyramidal neurons whereas others are mainly expressed in inhibitory interneurons. Lastly, we looked at possible confounds to *HTR mRNA* expression levels arising from our sample characteristics.

**Table 2:** The electrophysiological effects of the analyzed postsynaptic serotonin receptors (HTRs).

Receptor subtype	G-protein	Ion channel mediator	Physiological response in neuron
*Htr1A, Htr5A*	Gα_i_^(96,97,98)^	Increase potassium GIRK/Kir3 currents	Inhibition
*Htr2A, Htr2C*	Gα_q_^(98)^	Decrease potassium currentsIncrease nonselective cation current	Excitation
*Htr4, Htr6*	Gα_s_^(98)^	Decrease potassium currentIncrease nonselective cation current	Excitation

Information sourced from [96], [97], [98].

**Table 3:** Major and minor cortical cellular distribution of the analyzed serotonin receptors.

Pyramidal neurons	Interneurons
***Htr1A*** * ^(25, 26, 24, 27, 75)^	*Htr1A* ^(27, 75)^
***Htr2A*** * ^(25, 26, 24, 27,^ ^75)^	*Htr2A* ^(27,100, 99)^
***Htr4*** ^(45, 21, 46)^	***Htr2C*** ^(26, 37, 35)^
***Htr5A*** ^(26, 42, 41)^	***Htr6*** ^(48, 47)^

*Htr1A and *Htr2A* are highly co-expressed in prefrontal pyramidal neurons (24, 27).

Bold font denotes predominant cellular localization in cerebral cortex. Italic font denotes lesser cellular localization for receptors expressed in both excitatory and inhibitory neurons. Information sourced from [22], [25], [26], [27], [28], [36], [38], [42], [43], [46], [47], [48], [49], [55], [99], [100].

### *HTR1A* and *HTR5A:* Gα_i_-coupled receptors

Differences were observed with qPCR in the expression in one of the two inhibitory serotonin receptors in prefrontal cortex, as illustrated in **Figure 1**. We find that the mRNA for *HTR1A* does not change significantly across the age range evaluated (F(6,53)  = 0.6, p = 0.8). This developmental pattern contrasts with that for *HTR5A* mRNA expression which remains consistent across postnatal development, but then climbs sharply in adulthood (ANOVA: F(6,53)  = 2.9, p<0.02, Fisher LSD: p<0.02 adult vs. infant, neonate, school age, young adult), as illustrated in **Figure 1b**.

### *HTR2A* and *HTR2C:* Gα_q_-coupled receptors

**Figure 2:**
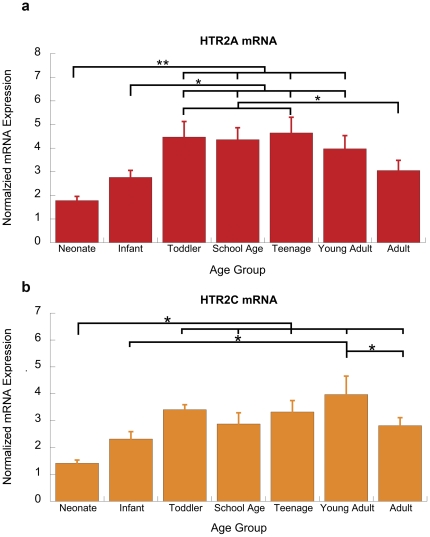
Developmental pattern of expression of Gα_q_-coupled serotonin receptors: *HTR2A* and *HTR2C*. (a) Normalized qPCR expression for *HTR2A* in human prefrontal cortex across postnatal development. The expression of *HTR2A* changes significantly across the developmental groups (F(6,53)  = 5.3, p<0.001) (b) Normalized qPCR expression for *HTR2C* in human prefrontal cortex across postnatal development. There is a significant developmental change in *HTR2C* expression (F(6,52)  = 4.7, p = 0.001). Asterisks show the significance levels of an ANOVA post-hoc Fisher LSD test (*p<0.05,**p<0.01).

There is strong developmental regulation of *HTR2A* mRNA in the human prefrontal cortex (F(6,53)  = 5.3, p<0.001). As illustrated in **Figure 2a**, this receptor shows a low level of expression in infancy, increases during the infant years, plateaus in the toddler to teenager period, and then declines to adult levels.

The similarly-coupled *HTR2C* appears to have opposing behavioural effects to the *HTR2A* [39], [40] potentially as a result of different cellular expression [36], [37], [38], [46], as illustrated in **Table 3**. Here, we find that in human prefrontal cortex *HTR2C* mRNA shows gradual developmental up-regulation (F(6,52)  = 4.7, p = 0.001). **Figure 2b** illustrates that prefrontal *HTR2C* expression reaches a highest expression during young adulthood.

### *HTR4* and *HTR6:* Gα_s_-coupled receptors

**Figure 3:**
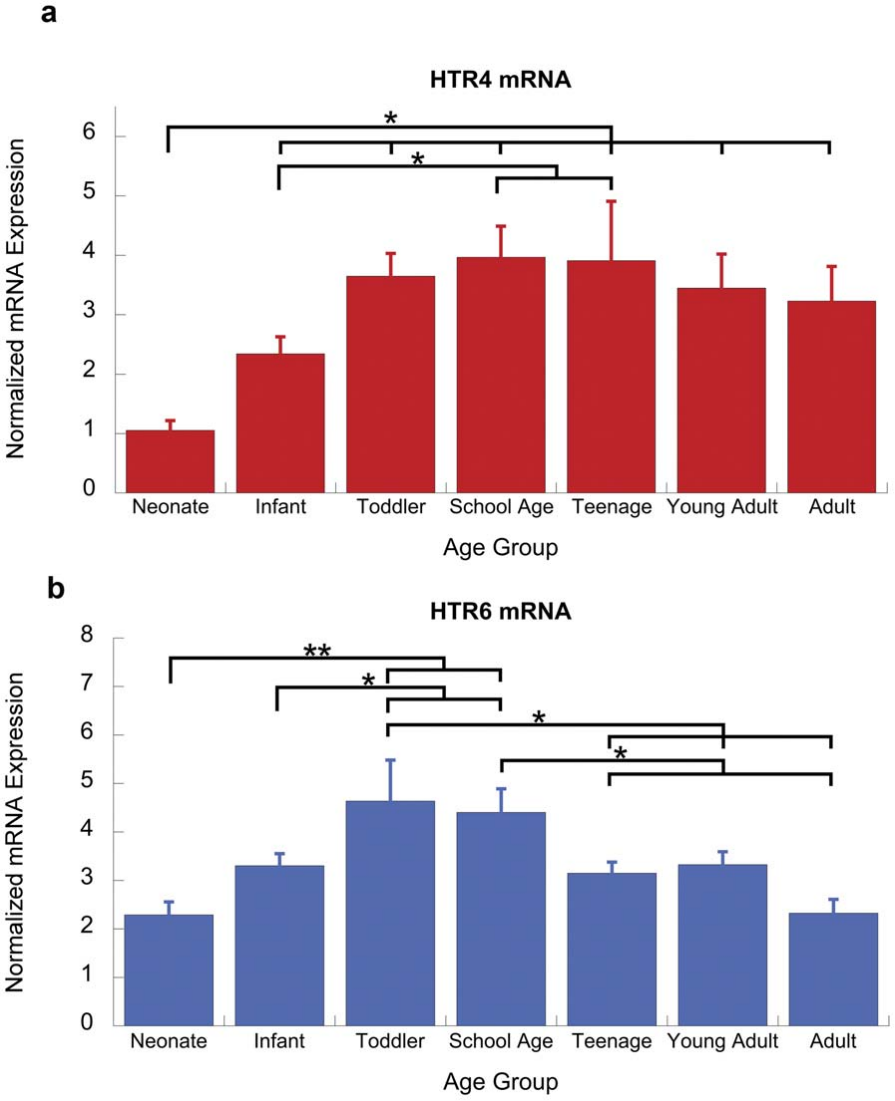
Developmental pattern of expression of Gα_s_-coupled serotonin receptors: *HTR4* and *HTR6*. (a) Normalized qPCR expression for *HTR4* in human prefrontal cortex across postnatal development. The expression of *HTR4* changes significantly across the developmental groups (F(6,50) = 4.6, p = 0.001). (b) Normalized qPCR expression for *HTR6* in human prefrontal cortex across postnatal development. There is also a significant developmental change in *HTR6* expression (F(6,50) = 4.9, p = 0.001). Asterisks show the significance levels of an ANOVA post-hoc Fisher LSD test (*p<0.05, **p<0.01).

*HTR4* mRNA is expressed in prefrontal pyramidal neurons [24], [46], [47] although this receptor results in weaker prefrontal excitation than *HTR2C* [24]. **Figure 3a** indicates that prefrontal *HTR4* mRNA is developmentally regulated (F(6,50)  = 4.6, p = 0.001), with peak expression during the school age and teenage years.

In contrast to the anatomical location of *HTR4*, *HTR6* has been previously suggested to have strong expression in inhibitory interneurons [48], [49]. Here, we find that there is a significant developmental regulation (F(6,50) = 4.9, p = 0.001), With the greatest *HTR6* mRNA expression in the toddler age group and a significant down-regulation after childhood (**Figure 3b** **)**.

### Relationship with inhibitory interneuron marker mRNA

Since *HTR2C* and *HTR6* are thought to be more strongly expressed in cortical interneurons, we examined whether their expression was related to developmental changes in common interneuron markers, such as parvalbumin (PV), calbindin (CB), somatostatin (SST) and cholecystokinin (CCK). As hypothesized, developmental changes in *HTR2C* and *HTR6* mRNA were significantly associated with individual interneuronal markers, but these two *HTRs* appear related to distinct interneuron markers. Developmental changes in *HTR6* mRNA were significantly matched with developmental changes in CB mRNA (R = 0.4, F(1,45) = 9.4. p = 0.004, β = 0.4). By contrast, developmental changes in *HTR2C* were significantly associated with changes in CCK (R = 0.6, F(1,47) = 28.5. p<0.001, β = 0.6). Despite some expression in interneurons [28], [55], [56], the developmental pattern of *HTR1A* expression could not be linked to any interneuron marker through regression. However, developmental changes in *HTR2A* mRNA were strongly associated with developmental changes in PV mRNA and CB mRNA (R = 0.9, F(2,46) = 98.2. p<0.001, β(PV) = 0.7, β(CB) = 0.5).

### Correlations with postmortem sample characteristics

Cortical pH was correlated with the expression of several serotonin receptors mRNAs (*HTR2A*: r = 0.51, p<0.001, *HTR2C*: r = 0.36, p = 0.007, *HTR6*: r = 0.29, p = 0.03, *HTR4*: r = 0.36, p = 0.01). PMI showed a significant negative correlation with *HTR2C* (r = -0.28, p = 0.04). There was no correlation between RIN values and any serotonin receptor. To establish if pH or PMI significantly changed the effect of age group, we performed ANCOVAs to compensate. When these correlated sample characteristics were co-varied, the results of results for all the *HTR* mRNAs remained statistically significant: *HTR2A*: F(6,52) = 3.7, p = 0.004, *HTR2C:* F(6,50) = 2.9, p = 0.02, *HTR6*: F(6,49) = 3.9, p = 0.003 *HTR4*: F(6,49) = 3.7, p = 0.004).

### Demographic effects other than age

None of the mRNAs encoding serotonin receptors varied significantly with gender. Caucasians did have a significantly increased expression of *HTR2A* mRNA compared to African Americans (t = −2.7,df = 57, p = 0.01). There was no universal demographic correlate in our study with these six HTRs.

## Discussion

We found large developmental changes in the expression of several individual serotonin receptors in human prefrontal cortex. While the mRNAs for two Gα_i_-coupled inhibitory *HTRs* have a relatively constant expression level across the life span, the mRNAs encoding two excitatory Gα_q_-coupled and two Gα_s_-coupled *HTRs* increase to higher levels during childhood and teenage years before declining to adult levels. However, some of these excitatory *HTRs* may be expressed predominantly in inhibitory interneurons, and thus may act to inhibit the prefrontal cortex. In support of this hypothesis, we found significant relationships between the developmental expression of certain *HTRs* and developmental expression of specific interneuron markers. Overall, a collective look at six prefrontal *HTRs* in terms of their neurophysiological effects and cellular localization suggests the possibility that there may be a balance in inhibitory and excitatory effects of serotonin across human postnatal development. Developmental changes in the expression of individual *HTRs* in the normal human prefrontal cortex from infancy to adulthood give insight into how the brain normally develops and provide a reference to compare changes which may occur in response to genetic or environmental circumstances which would alter one or more specific subtypes of serotonin receptor.

### Model for the developmental balance of the expression of excitatory and inhibitory HTRs

**Figure 4:**
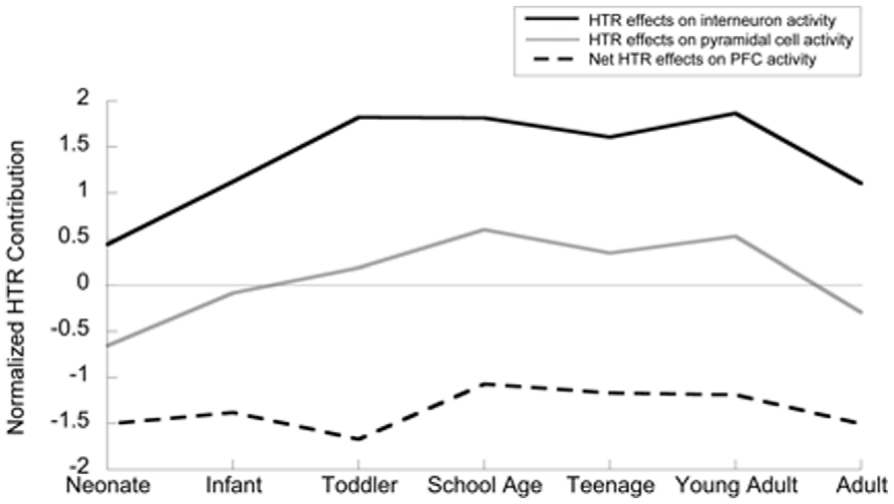
Working model to illustrate the balanced developmental expression of *HTRs* with excitatory effects on prefrontal cortex and those with inhibitory effects. The complex cellular localization of the HTRs and their different excitatory or inhibitory effects makes it difficult to conceptualize how the developmental changes in their expression would affect the prefrontal cortex. Several assumptions were required in order to formulate a working model that attempted to synthesize the potential consequences of these developmental changes. The mRNA expression of each serotonin receptor was normalized to its individual peak expression which was given the value of one. Receptors on which serotonin has an excitatory effect were assigned a positive contribution value while those receptors on which serotonin has an inhibitory effect were assigned a negative value. The net HTR contribution to interneuron activity predicted by this model is plotted in black and was calculated as follows: (*HTR2A* + *HTR2C* + *HTR6* – *HTR1A*). The net HTR contribution to pyramidal neuron activity predicted by this model is plotted in gray and was calculated as follows: (*HTR2A + HTR4* - *HTR1A* - *HTR5A*). The overall effect of serotonin receptors on the output from the prefrontal cortex was based on the predominant localization of each receptor. This net prefrontal effect is illustrated by the dashed line which takes into account that excitation on inhibitory interneurons would likely result in inhibition of the firing of the pyramidal cells responsible for output. Note this working model suggests a lack of a large developmental change in the predicted overall net effect of *HTRs* on the prefrontal cortex, despite an overall trend towards increased expression of the excitatory serotonin receptors within the first decade of life. However, the 5HTR subtypes responsible for maintaining this balance appear to change.

We found strong developmental regulation of the individual *HTR* mRNAs, in particular the Gα_q_-coupled and Gα_s_-coupled *HTRs*. By considering the possible excitatory or inhibitory effect of our measured HTRs, as well as their proposed cellular locations, we produced the model illustrated in **Figure 4** **.** We calculated the proposed interneuron contributions of the HTR receptors (*HTR2A* + *HTR2C* + *HTR6* – *HTR1A*) as well as the pyramidal cell contributions (*HTR2A + HTR4* - *HTR1A* - *HTR5A*). By taking into account that excitation of inhibitory cells will result in an inhibition of pyramidal cell firing, we were able to propose a net effect (dotted line in Figure 4) on the prefrontal cortex. This overall effect was seen to be balanced throughout the course of prefrontal cortical development. Overall, serotonin signalling may have an increased saliency in the first 5–10 years of life since the expression of most *HTR mRNAs* increase during this developmental period. In order to reduce the complexity of the system, the model makes several assumptions. It only includes 6 of the 14 known HTR receptors and does not take into account variations in receptor densities and possible interactions of second messenger systems. However, the model provides a useful framework for evaluating the possible net effects of serotonin over the course of prefrontal cortical development.

### Developmental expression of Gα_i_-coupled HTRs in human prefrontal cortex

#### *HTR1A:* Perspective and clinical relevance

Our finding of relatively constant *HTR1A* expression across human development is consistent with the results of a previous developmental investigation in human *postmortem* tissue [57]. *HTR1A* has been a focus of myriad human *postmortem* and imaging studies in adults. Work examining *HTR1A* binding in prefrontal cortex suggests that such Gα_i_-coupled receptors are protective against anxiety in controls [32] and are reduced in people with social anxiety [31]. Interestingly, there appears to be an increase in *HTR1A* expression and binding in schizophrenia [26], [58], [59], [60].

The importance of maintaining constant expression of *HTR1A* in prefrontal cortex during development should not be underestimated, since conditional loss of the forebrain *Htr1A* receptor during development leads to life-long abnormal anxiety behaviour [61], [62]. Of note, *HTR1A* appears to be regulated by early life experience in rodents and primates [41], [63]. Human *HTR1A* is highly expressed in superficial layers of prefrontal cortex [64]. Analysis of *Htr1A* expression and function in superficial prefrontal cortex of rodent suggests that healthy animals have relatively stable levels of this key inhibitory receptor [41]. However, a careful examination of the *in situ* data [41], in the context of previous neurophysiological examination [22], [23], suggests that *Htr1A* may have varying developmental mRNA expression patterns in the superficial and deep cortical layers. An apparent lack of change in *HTR1A* mRNA expression in the development of the prefrontal cortex by qPCR analysis may obscure possible opposite developmental changes in the superficial and deeper cortical layers [22], [41]. Despite a link to PV-containing interneurons [30], [55], [56], [65], *HTR1A* mRNA levels did not display a relationship through regression with any mRNA marker of inhibitory neurons.

#### *HTR5A:* Perspective and clinical relevance

The other Gα_i_-coupled receptor we examined, *HTR5A*, is less fully understood. We found its expression levels remain relatively constant throughout development but it shows an unexpected and fairly large increase in expression in adulthood. Stimulating *HTR5A* may be underappreciated as a clinical target to protect the brain under stressful conditions. A genetic deletion study in mouse suggests that knocking out *Htr5A* produces abnormal behavioural effects on stressful tests and inappropriate responses to novel situations [45]. Interestingly, a close family member *(Htr5B)* has recently been shown to be upregulated by the developmental stress of social isolation after weaning [66].

### Developmental expression of Gα_q_-coupled HTRs in human prefrontal cortex

#### *HTR2A:* Perspective and clinical relevance

The *HTR2A* receptor is the second most widely expressed serotonin receptor in the prefrontal cortex after *HTR1A* and is also predominantly expressed in pyramidal neurons [25], [26], [27], [28], [67]. In rodents, the excitatory effects of cortical *Htr2A* are thought to promote anxiety [33] and impulsivity [40]. In adult humans, *HTR2A* binding is elevated in prefrontal cortex of subjects with mood disorders [35] and also has been correlated with personality risk factors for affective disorders, such as neuroticism [68], [69]. *HTR2A* antagonism contributes to the efficacy of medicines used to treat schizophrenia to the extent that it may be a defining feature of the atypical antipsychotic medicines [70]. However, it is controversial whether there are changes in *HTR2A* expression or binding in schizophrenia [26], [59], [71], [72], [73]. Yet, medicines that antagonize *HTR2A* improve the symptoms of anxiety and depressive disorders in patients who did not improve on other treatments [74], [75], [76], [77]. In rodents, genetic deletion of cortical *Htr2A* diminished anxiety levels [33], and pharmacological modulation of *Htr2A* within the prefrontal cortex has bidirectional effects on impulsivity with stimulation increasing and antagonism decreasing impulsivity [40]. Rodent work has also shown strong neurophysiological effects of this receptor in deep layers of cortex in the first two weeks of postnatal development [22], [23] and reduced effects in adulthood [78], likely due to the competing inhibitory effects of *Htr1A* which is highly co-expressed in cortical pyramidal neurons [25], [28]. Recent work suggests that early stress exposure may increase the functional effects of *Htr2A* in adulthood with negligible changes in receptor expression, binding, or sensitivity [78], suggesting potential elevation of downstream signalling.

*HTR2A* mRNA also shows a remarkably strong positive relationship with the interneuron markers PV and CB, both of which show a protracted increase during the first decade of human life [7]. Of note, the up-regulation of *HTR2A*, PV, and CB mRNA mirrors the increase in gamma oscillation power which is found during development [79]. PV and CB are found primarily in inhibitory neurons which are electrophysiologically classified as fast-spiking and which synapse onto the cell bodies of excitatory neurons [80]. *Htr2A* has been suggested to modulate cortical gamma oscillations [9], [30] at least in part through its effects on fast spiking interneurons [30], [56].

#### *HTR2C:* Perspective and clinical relevance

*HTR2C* is thought to have effects opposite to those of *HTR2A* within prefrontal cortex. This difference may be due to the fact that *HTR2C* is expressed in inhibitory interneurons [36], [37], [38] and not in pyramidal neurons [46], while *HTR2A* is predominantly expressed in pyramidal neurons with a minority of its expression in interneurons [28]. Here, we show that the developmental changes in *HTR2C* are linked to developmental changes in the interneuronal marker, CCK. This relationship suggests that as CCK interneurons mature they may increase *HTR2C* expression and become more responsive to serotonin. Work in rodents suggests that greater prefrontal *HTR2C* tone would act to decrease impulsivity, since impulsivity is increased either by genetic deletion of *Htr2C* [39] or by its pharmacological antagonism within prefrontal cortex [40].

### Developmental expression of Gα_s_-coupled HTRs in human prefrontal cortex

#### *HTR4:* Perspective and clinical relevance

The *HTR4* receptor is found in prefrontal cortical pyramidal neurons [24], [46], [47] and appears to have excitatory effects within prefrontal cortex [22], [24]. There is evidence that *HTR4* agonists improve cognitive functioning in rodent and primate models [81], [82]. Our findings are broadly consistent with rodent studies suggesting that there is gradual up-regulation of this receptor during development [83]. The surface expression and function of *HTR4* are thought to be regulated by the S100 family member p11 [84], which has been implicated in mood disorders and suicide [85]. The *HTR4* receptor gene also has a polymorphism which has been linked to schizophrenia in a Japanese population [86].

#### *HTR6:* Perspective and clinical relevance

*Htr6* appears to be more strongly expressed in inhibitory interneurons [48], [49] than in pyramidal neurons [46]. We found that developmental changes in *HTR6* expression follow an inverted-U shaped pattern, with an initial increase followed by a decrease. This expression profile mirrors that for CB expression in our cohort, which corresponds to the proposed interneuron localization of *HTR6*. Stimulation of this receptor has been suggested to have anxiolytic [49], [87] and anti-depressant effects [88]. Yet *HTR6* antagonism is an unusual feature of the atypical antipsychotic clozapine [89]. Indeed, pharmacologically antagonizing *HTR6* acts to enhance cognition [51]. In our cohort, the expression of *HTR6* peaks at the toddler age group before decreasing into adulthood, thus drugs acting on this receptor may produce exaggerated effects if administered in childhood.

### A balance seemingly tipped towards inhibition and potential mechanisms of dysregulation

Quantification of the normalized expression of two serotonin receptors likely to have net excitatory effects on the prefrontal cortex (*HTR2A* and *HTR4*) based on the available evidence, and four likely to have net inhibitory effects (*HTR1A, HTR2C, HTR5A, HTR6*), suggests that the effect of serotonin through these receptors may remain balanced in the inhibitory range across development and in adulthood. This prediction of a net inhibitory cortical response to serotonin is supported by *in vivo* studies of the cortical effects of raphe stimulation in adult rodents [11], [25], [29]. The overall effect of serotonin on the cortex is complicated by the localization of a number of these receptors on inhibitory interneurons (*HTR2C, HTR6, and to a lesser extent HTR1A, HTR2A*). This is shown by the widespread relationship between developmental changes in GABAergic interneuron marker mRNA expression and those of *HTRs* expression. In particular, there is a statistical relationship demonstrated through linear regression for *HTR2A* and *HTR6* mRNA with the interneuron marker CB, in addition to a relationship between *HTR2C,* and the interneuron marker CCK.

The hypothesized balance between excitation and inhibition for this group of receptors may be disrupted during development when the expression or function of individual *HTRs* are changed by polymorphisms, early life stress, or developmental exposure to serotonergic medicines. For example, the developmental trajectory of *HTR2A* suggests that the period of increased *HTR2A* mRNA expression from childhood through the teenage years may be a time when the brain would be particularly vulnerable to increased developmental *Htr2A* expression resulting from early maternal deprivation [78], [90]. If unchecked by the highly co-expressed inhibitory *Htr1A* [25], [28], or by the excitatory *Htr2C* expressed in inhibitory neurons [35], [36], [37], [38], [39], such changes would be hypothesized to lead to greater impulsivity [39], [40] particularly under stressful conditions which would increase serotonin release [1], [11], [91]. Changes to the expression or function of *HTRs* have been shown to occur as a result of genetic polymorphisms [92], [93], [94], early life experience [41], and developmental pharmaceutical exposure [15], [95]. Disrupting a normal balance in the effects of serotonin on the prefrontal cortex during a vulnerable developmental period may result in abnormal emotional regulation, cognition, and behaviour in the short term. Furthermore, the trophic effects of serotonin on maturing neurons [18], [19], [20], [21], suggests that dysregulation of an individual *HTR* may have lasting repercussions for adult prefrontal cortical structure and function.

## Supporting Information

Table S1 Demographic details of cases used in this study. Abbreviations: m, male; f, female; PMI, post-mortem interval defined as interval between death and freezing of the brain; AA, African American; C, Caucasian; RIN, RNA integrity number; SIDS, sudden infant death syndrome; MVA, motor vehicle accident; SVCS, Superior Vena Cava Stenosis; ASCVD, Arteriosclerotic Cardiovascular Disease; HASCVD, Hypertensive Arteriosclerotic Cardiovascular Disease.
(DOCX)
