# Supplementary material for: Serotonin Receptor Expression in Human Prefrontal Cortex: Balancing Excitation and Inhibition across Postnatal Development
Source: PLoS One. 2011 Jul 29;6(7):e22799. doi: 10.1371/journal.pone.0022799 (PMC3146513; doi:10.1371/journal.pone.0022799)
Supplement: Table S1 — Demographic details of cases used in this study. Abbreviations: m, male; f, female; PMI, post-mortem interval defined as interval between death and freezing of the brain; AA, African American; C, Caucasian; RIN, RNA integrity number; SIDS, sudden infant death syndrome; MVA, motor vehicle accident; SVCS, Superior Vena Cava Stenosis; ASCVD, Arteriosclerotic Cardiovascular Disease; HASCVD, Hypertensive Arteriosclerotic Cardiovascular Disease. (DOCX) [file pone.0022799.s001.docx]

**Table S1:** Demographic details of cases used in this study

| Group | PMI (hours) | Gender | Age (years) | pH | Race | average RIN | cause of death |
| --- | --- | --- | --- | --- | --- | --- | --- |
| Neonate | 28 | m | 0.21 | 6.6 | AA | 8.5 | SIDS |
| Neonate | 11 | m | 0.15 | 6.9 | C | 8.3 | congenital heart defect |
| Neonate | 17 | m | 0.15 | 6.6 | AA | 8.7 | SIDS |
| Neonate | 19 | f | 0.18 | 6.5 | C | 7.1 | asphyxia |
| Neonate | 25 | m | 0.19 | 6.5 | AA | 8 | asphyxia |
| Neonate | 27 | f | 0.16 | 6.5 | AA | 7.9 | pneumonia |
| Neonate | 27 | m | 0.11 | 6.5 | AA | 7.9 | asphyxia |
| Neonate | 24 | f | 0.24 | 6.7 | AA | 7.7 | positional asphyxia |
| Infant | 14 | f | 0.25 | 6.5 | AA | 8.8 | SIDS |
| Infant | 22 | f | 0.52 | 6.8 | AA | 8.4 | SIDS |
| Infant | 18 | f | 0.48 | 6.5 | AA | 6.5 | SIDS |
| Infant | 9 | m | 0.38 | 6.5 | AA | 7.2 | SIDS |
| Infant | 18 | m | 0.91 | 6.9 | AA | 8 | SIDS |
| Infant | 24 | m | 0.52 | 6.7 | AA | 8.2 | accident/asphyxia |
| Infant | 5 | m | 0.39 | 6.8 | AA | 8.6 | asthma |
| Infant | 21 | f | 0.67 | 6.6 | AA | 5.9 | SIDS |
| Infant | 22 | m | 0.33 | 6.5 | C | 8 | bronchoneumonia |
| Infant | 10 | f | 0.91 | 6.4 | AA | 6.6 | bronchiolitis |
| Infant | 19 | m | 0.32 | 6.4 | C | 6.7 | asphyxia suffocation |
| Infant | 27 | m | 0.35 | 6.7 | C | 8.1 | myocarditis |
| Infant | 18 | m | 0.82 | 6.65 | AA | 7.3 | hypothermia |
| Toddler | 24 | f | 1.58 | 6.9 | C | 7.8 | myocarditis |
| Toddler | 18 | m | 4.64 | 6.9 | C | 7 | accident |
| Toddler | 11 | f | 2.21 | 6.9 | AA | 7.4 | meningitis |
| Toddler | 22 | f | 2.45 | 6.7 | AA | 7.6 | no anatomical cause |
| Toddler | 20 | f | 2.47 | 6.5 | C | 5.7 | MVA |
| Toddler | 13 | m | 2 | 6.89 | AA | 6.9 | cardiac arhythmia |
| Toddler | 27 | m | 2.19 | 6.6 | AA | 7.6 | asthma |
| School age | 16 | m | 12.42 | 6.8 | C | 8.2 | drowning |
| School age | 18 | m | 7.84 | 6.8 | AA | 7 | accident |
| School age | 18 | f | 12.98 | 6.9 | C | 7.8 | accident |
| School age | 12 | f | 8.92 | 6.4 | C | 6.7 | cardiac arhythmia |
| School age | 12 | f | 11.54 | 6.4 | C | 7.3 | asthma |
| School age | 20 | f | 8.14 | 6.8 | C | 7.6 | asphyxia |
| School age | 5 | m | 8.01 | 6.8 | AA | 8.2 | cardiac arhythmia |
| Teenage | 16 | m | 17.49 | 6.7 | C | 6.5 | accident |
| Teenage | 13 | m | 15 | 6.8 | AA | 6.2 | accident |
| Teenage | 16 | m | 17.69 | 6.8 | AA | 8.2 | accident |
| Teenage | 25 | m | 17.05 | 6.7 | C | 7.5 | drowning |
| Teenage | 19 | m | 17.38 | 6.84 | C | 6.8 | accident |
| Teenage | 16 | f | 16.68 | 6.81 | C | 7.6 | multiple injuries |
| Teenage | 20 | f | 16.34 | 6.6 | C | 6.7 | multiple injuries |
| Young adult | 32 | f | 25.1 | 6.54 | C | 6.8 | pulmonary embolism |
| Young adult | 12 | m | 22.5 | 6.75 | C | 5.5 | MVA |
| Young adult | 16 | f | 25.38 | 6.73 | C | 8.3 | accident |
| Young adult | 4 | m | 22.92 | 6.84 | AA | 8.2 | ASCVD |
| Young adult | 13 | f | 21.93 | 6.96 | C | 7.8 | MVA |
| Young adult | 18 | m | 20.14 | 6.5 | AA | 7.2 | accident |
| Young adult | 7 | m | 21.97 | 6.25 | AA | 6.9 | obesity |
| Young adult | 7 | m | 24.93 | 6.92 | C | 8.4 | MVA |
| Young adult | 14 | f | 23.62 | 6.57 | AA | 8.1 | asthma |
| Adult | 18 | m | 46.18 | 6.75 | AA | 7.8 | accident |
| Adult | 18 | m | 42.94 | 6.49 | C | 7.3 | accident |
| Adult | 13 | m | 35.99 | 6.73 | C | 8 | coronary artery disease |
| Adult | 8 | m | 38.63 | 6.37 | AA | 7.6 | ASCVD |
| Adult | 12 | m | 47.44 | 6.56 | C | 6.4 | ASCVD |
| Adult | 12 | f | 48.7 | 6.12 | C | 5.2 | ASCVD |
| Adult | 19 | f | 38.42 | 6.98 | AA | 7.6 | HASCVD |
| Adult | 7 | f | 49.22 | 6.78 | AA | 7.4 | cirrhosis of liver |
